# Acceptability of a practical geriatric assessment intervention with older adult cancer survivors and community health workers/promotoras: a qualitative investigation

**DOI:** 10.1007/s00520-026-10473-9

**Published:** 2026-02-23

**Authors:** Alex J. Fauer, Sandra Calderon, Quynh Vo, Miriam Hernandez, Angela Usher, Chad Y. Han, Frederick J. Meyers, Diana L. Miglioretti

**Affiliations:** 1 Betty Irene Moore School of Nursing, University of California Davis, 2450 48th St., Ste 2600, Sacramento, CA 95817 USA; 2 Comprehensive Cancer Center, University of California, Davis, Sacramento, CA 95817 USA; 3 Visión y Compromiso, Los Angeles, CA USA; 4 College of Nursing and Health Sciences, Flinders University, Adelaide, SA Australia; 5 Department of Public Health Sciences, University of California, Davis, Davis, CA USA

**Keywords:** Cancer, Supportive care, Geriatric oncology, Community engagement, Community health worker, Promotoras de salud

## Abstract

**Purpose:**

Determine the acceptability of a community-based, practical Geriatric Assessment (GA) intervention among community health workers (Promotoras) and cancer survivor key informants. Promotora-delivered health assessment, education, and social support models of care are feasible and effective in chronic disease management, but more evidence is needed in the cancer survivorship context. Promotoras function as a bridge, connecting community-based services with populations with historically limited healthcare engagement.

**Methods:**

Descriptive qualitative design. We held focus groups with promotora key informants and interviews with older cancer survivor consultants, between January and February 2024. Researchers developed a semi-structured interview guide, informed by conceptual models and an implementation framework, to facilitate perceptions of promotora-guided interventions, GA, and cancer survivorship. Researchers performed content analysis with deductive and inductive techniques on transcribed interviews.

**Results:**

Acceptability of the GA intervention was described by promotoras and cancer survivor consultants within three distinct categories: The GA intervention employs a motivator to help address survivorship complexities; the GA intervention plans for impaired ADLs and IADLs in survivorship; the GA intervention integrates promotoras with clinical oncology care.

**Conclusion:**

The proposed implementation of a practical GA intervention by promotoras was deemed acceptable. The findings provided essential contextual data to ensure that the pilot, practical GA study can establish feasibility and preliminary efficacy.

**Supplementary Information:**

The online version contains supplementary material available at 10.1007/s00520-026-10473-9.

## Introduction

In 2022, there were an estimated 18.1 million cancer survivors in the United States (US) [1], and by 2040, 73% of cancer survivors are expected to be age 65 or older (i.e., older adults) [2]. Despite advances in survival rates, most older cancer survivors are at greater risk for health challenges following diagnosis for treatment for cancer including functional decline and troubling symptoms [3]. In cancer care, the geriatric assessment (GA) intervention evaluates age-related vulnerabilities, and helps improve survival via early identification of treatment toxicity in older adult cancer survivors [4, 5]. Although GA is the recommended standard of care for older adults with cancer receiving curative therapy [6], it is not a routinely delivered assessment for cancer survivors. A “comprehensive” GA is time-intensive and requires multidisciplinary coordination with geriatric oncology specialists [6]. Instead, a “practical” GA has been considered more suitable [6, 7], but there is limited evidence of its efficacy in community settings [8]. Most GA clinical interventions have been implemented in well-resourced, academic oncology settings without diverse patient catchment areas [9, 10]. More evidence is needed to tailor practical GA for specific cancer survivor populations instead of implementing a universal approach.

Our investigative team is developing a practical GA intervention in collaboration with promotoras de salud (i.e., “promotoras”, community health workers), who are bilingual in English/Spanish. Promotora-delivered health assessment, education, and social support interventions have been shown to be feasible in chronic disease contexts [11, 12], but not specifically for cancer survivorship care. Promotoras work in association with the community health care system to provide health education and information, deliver informal counseling and advocate for individual and community health needs [13]. Promotoras bridge community-based services with populations that have historically limited healthcare engagement [14], which may lead to transferrable insights for cancer survivorship given the barriers to GA in community settings.

This study aims to understand the acceptability of a community-based, practical GA intervention, which is intended to be delivered by promotoras. In this study, promotoras describe suitable strategies to assist older cancer survivors with their personal health goals and outcomes. The qualitative analysis evaluates how promotoras strategies and beliefs align with existing geriatric oncology and survivorship care.

### Conceptual model

**Fig. 1:**
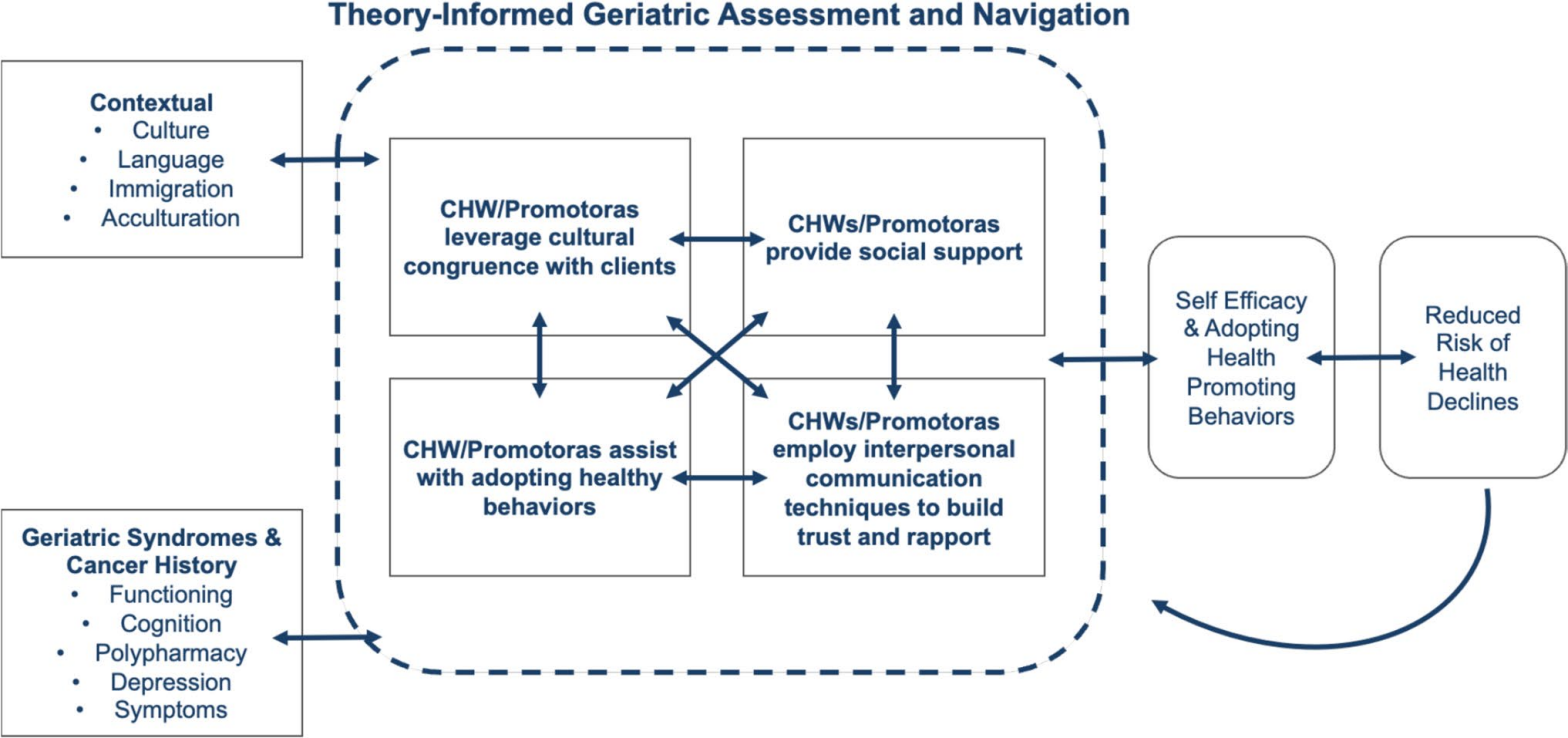
Conceptual model

We identified an existing conceptual model of collaborating with promotoras in community-based research from Katigbak et al. [15]. Then, we adapted the Katigbak model with concepts from updated consolidated framework for implementation research [16]. Promotoras represent the implementation facilitator and follow a collaborative process with older adult cancer survivors to improve survivor self-efficacy and social support [17]. In our broader research, we intend for the promotoras to leverage knowledge of the inner setting (e.g., health system) and outer setting (e.g., community setting resources) to navigate the older cancer survivor to recommended care. The conceptual model and implementation framework both informed acceptability topics for our semi-structured interview guide (see Fig. 1).

## Methods

### Design and approach

The research team conducted a descriptive qualitative study with semi-structured focus groups and interviews [18]. A descriptive approach was chosen to pragmatically understand (a) the acceptability of a planned community-based, practical GA intervention, which is intended to be delivered by promotoras. The study launched in December 2023 and recruitment of participants concluded in February 2024. As interview and focus group research, this project was deemed “Exempt” from human subjects review by the university’s Institutional Review Board (IRB). Oral consent was obtained from each participant. The investigative team used the Standards for Reporting Qualitative Research (SRQR) checklist to format this article [19].

### Sample and setting

We enrolled a convenience sample of promotoras and, separately, older adult cancer survivors as consultants. All participants received a $40 e-gift card incentive. To ensure participant confidentiality, all identifying details have been removed from this paper.

The study’s community partner, Visión y Compromiso (VyC), identified 19 promotoras from their California-based network who expressed interest in cancer, geriatrics, or caregiving, and agreed to participate in the research. Eligibility criteria for cancer survivor participants included being 65 years of age or older, having a prior cancer diagnosis, and being able to speak either English or Spanish. The interviews with cancer survivors were conducted in English. We distributed electronic health record (EHR) digital advertisements to a random sample of 50 local cancer survivors that met eligibility criteria who received care at the study institution: a comprehensive cancer center in California. The study setting context is noteworthy. Currently, there is no dedicated geriatric oncology clinic to conduct GA. Older age groups represent most of the case load for the comprehensive cancer center’s catchment area.

### Measurement

We developed a semi-structured interview guide with open-ended questions to elicit perspectives from promotoras and cancer survivors about post-treatment care and aging concerns. The questions were informed by the promotora intervention conceptual model, well-documented health needs of older adult cancer survivors post-treatment, including frailty, polypharmacy, symptom burdens, and caregiver needs [20–23]. The interview guide is included in Supplemental File 1. Prompts for the promotoras, in English, included but were not limited to, *“What resources have you provided for an older adult with a history of falls? What is your process to provide emotional support for an older person who is under a lot of stress, or is alone?*” We re-phrased questions for the survivor participants, including, but not limited to, *“What has been helpful for you to take care of yourself and any symptoms you may have experienced? What has been helpful for your family caregiver? If you could imagine an ideal clinic for older cancer survivors, what would be the best type of role to interact with you?”.*

### Procedure

Focus groups explored the promotoras’ caregiving experiences and the types of support they need in their roles. The focus groups lasted 75 to 90 min, held virtually with a teleconferencing system (Zoom), and conducted primarily in Spanish with English translation completed by a bilingual study team member. Our total promotora sample (*N* = 19) met theoretical and practical standards to achieve ideal saturation of collected, descriptive qualitative evidence [24].

We purposively enrolled a convenience sample of 3 older cancer survivors in the local community as consultants, with interviews held in-person at a non-clinical office location on the study institution’s campus. Interviews lasted 60 to 75 min and focused on survivors’ perceived usability of practical GA by promotoras for cancer survivorship issues, such as physical function and mental health. One interview was held with a group of two participants to accommodate and encourage interaction without veering from the semi-structured protocol. One interview was conducted one-on-one with the interviewers and an older adult, cancer survivor participant. In line with Morse’s guidance on qualitative sample adequacy for exploratory work, a small consultant sample (*N* = 3) was appropriate for obtaining preliminary usability insights rather than achieving saturation [25].

Audio-recordings from both interview formats were transcribed verbatim using a third-party vendor, removing any identifying information.

### Data analysis

Three researchers conducted the analysis (A.F., S.C., and Q.V.). Deductive and inductive content analysis was used to analyze the data [26, 27]. Preliminary analysis began with debrief meetings after each interview. Debriefing allowed the researchers to improve the coding approach and prompts in response to evolving study findings. In-depth analysis, beginning with deductive approaches, began as soon as audio-recordings were transcribed. Two to three researchers independently read through each transcript, listing key points organized by domains from the interview guide and conceptual model. Upon completion of deductive analysis, we analyzed the data again using an inductive approach to identify nuances within the text. The researchers refined the dominant categories and created a codebook. In total, the researchers met four times from April 2024 to August 2024. The researchers used the finalized codebook to code the data from the interviews using the qualitative management software Dedoose (Los Angeles, CA, USA).

## Results

### Interview and focus group findings

Three qualitative content categories revealed how older adult cancer survivors and promotoras perceive the proposed implementation of a practical GA intervention: The GA intervention (1) employs a motivator to help address survivorship complexities, (2) plans for impaired ADLs and IADLs in survivorship, and (3) integrates promotoras with clinical oncology care.

### The GA intervention employs a motivator to help address survivorship complexities

Survivors expressed needs for a motivator who comprehends, and empathizes with, the challenges faced by older cancer survivors navigating resources alone, concerns about lingering symptoms, and promoting their health. Previously, one survivor mentioned their interest in supportive care and survivorship programs diminished when the provider was not attentive to their individual needs and concerns. The proposed GA intervention serves as a platform for survivors to engage with a promotora who has lived experience as a primary caregiver and specific training on the associated challenges of aging in cancer survivors.

Yeah, I think demeanor matters a lot. I think if somebody can convey that we’re in this together that’s a huge motivator. I think the fact that their knowledge shows that they know what they’re doing. What else? They realize that you’re human, that you don’t always do what you should be doing, but you can pick it up and continue along. Why did I respond so well to that physical therapist? It’s probably just ’cause she was so good. She just radiated competence. – Cancer Survivor 1

While promotoras and survivors alike reported awareness that clinical programs exist for symptom management, it was implied that physicians often do not initiate discussions about mental health needs. Promotoras suggested survivors who could engage in mental health conversations with their providers would report increased validation and strengthened therapeutic relationships. Promotoras expected to console cancer survivors about potential depressive symptoms, anxiety, and symptoms of trauma.

When they’re diagnosed or they’re given the cancer diagnosis, the person changes their way of thinking, changes their behavior, changes in mood swings, so they get upset, they get mad, they don’t want to go outside, they don’t want to do exercise so that’s like at the beginning where they just, they were able to find out this diagnosis. But they don’t know what it’s going to take them to. So, I learned that it goes by stages. At the beginning they feel emotional so the first thing I was able to get was behavioral services.– Promotora

### The GA intervention plans for impaired ADLs and IADLs in survivorship

In the interviews, preserved physical function was considered essential to maintaining independence as participants aged. Survivors reported understanding that systemic cancer treatments may reduce mobility, balance, and strength. Potential health concerns represent, for example, reduced performance of instrumental activities of daily living (IADLs) and ADLs, increased risk of falls, and decreased physical activity levels. The promotoras involvement in the GA intervention would provide cancer survivors with a supportive space to openly discuss physical activity, impacting ADL/IADLs and health-related quality of life topics that were previously difficulty to address with healthcare providers.

It went from that to the doctor saying, ‘You need to exercise, you need to do this.’ So, he gave us a list of activities, like a 40-minute walk. They were [encouraging us] to do so but the problem is when no one is there or they don’t have anybody to do, even the walk. They don’t have a relative, they don’t have a representative, they don’t have an exercise provider, that’s when they stop. – Cancer survivor 2

In the last two years I’ve lost 60 pounds I didn’t wanna lose ‘cause I was diagnosed with radiation enteritis. I’ve gained 10 pounds [laughter] since July, so it’s coming back a little bit, so that’s good. I was swimming six days a week or seven days a week and doing all those kinds of things, and then I’ve had some other neurological things starting to happen as well as some dermatology things that prevented me from being in the pool and stuff.—Cancer survivor 3

Most cancer survivor participants acknowledged the accessibility of physical therapy (PT) and occupational therapy (OT) to address aging concerns, while formal mental health care was less accessible. Additional resources survivors expressed a desire for include nutrition programs, cognitive exercises and personal training. Some participants emphasized that the implementation of mandatory participation in these programs would be beneficial.

If there was a mandatory nutrition program, that probably would have been helpful. I don’t know if you could make it mandatory. I think that actually would have been helpful. – Cancer Survivor 1

Survivors expressed their concerns regarding the inadequate assistance and referrals to resources offered by their physician and healthcare teams. While clinicians often recommend and motivate patients to adopt healthy practices (e.g., engaging in regular walks) additional peer support is usually welcomed to combat lack of motivation and physical limitations.

I think there’s this resistance to— particularly nutrition is probably where I mostly fall down. Even in the exercise—I assume this is me personally, but I definitely don’t always do what I should be doing. I expect many people have that, and something along the way, if there’s some way to motivate or a social setting or a group or a something that everyone’s trying to move in the same direction at the same time is—for any of these aspects, could be very helpful. – Cancer Survivor 2

### The GA intervention integrates promotoras with clinical oncology care

Promotoras perceived the GA intervention strongly from prior caregiving experiences for older family members. Often the promotoras encouraged the care recipient to engage in exercise, activities outside the home, and were aware of relevant signs and symptoms that warranted communication with the care team.

I saw that with my mother…she died at 91 years old and the first thing she would say as soon as she got up, “Let’s do exercise.” She was always doing exercise. It was only when her anemia would drop a lot to the point that she needed blood that she would say, “Today I am not doing exercise, I am tired.” But as soon as she would feel better, she’d say, “Let’s go around the world.” And they feel more motivated when you can take them out for walks, motivating them to keep going. They can have the strength to live a little longer. Like I said, my mom died, aside from leukemia she also had cardiac deficiency and other health problems. – Promotora

Promotoras noted challenges with communicating acute issues (e.g., toxicities) with the care team, especially a Hispanic or Latino caregiver perspective. This was a notable concern for one promotora, who considered themselves as a trained, competent linkage between an underrepresented community and the health system. It is common that providers state they don’t have enough time or resources to assess or navigate survivorship resources to patients.

My father is a cancer survivor. That was three years ago. Regarding trust, what my colleague was saying is very important. This happens even with close relatives like my father. Right at the moment when they found it, thanks to my experience as a promotora, I was able to help him so he wouldn’t have to go back to the hospital or the emergency room. I was with him the whole time. It’s important to have trust and make him realize it. – Promotora

The promotoras described familiarity with community resources to enhance independence and social engagement for older adults, although not solely intended for cancer survivors. These programs are often organized and planned by local non-profit and government agencies. Promotoras considered the GA intervention can act as the starting point to facilitate access to resources that support the cancer survivors personal goals and needs.

It’s here in [redcated], there are classes, I would take my mom and she was very shy. She would say, “I’d rather do my exercise at home.” But she went on two occasions and said, “No, it’s better at home, I’ll do my exercise.” But there are places where you can take the older adults to motivate them to do exercise, play games with other people and they can talk with other people, and it can be very motivating.. – Promotora

If you make a call to the Senior Lot project, it gives you all the resources that are related to people who are 60 or older. These are food resources, community, clothes, even to fill out applications for [public insurance], if they don’t know their insurance, they guide them. – Promotora

## Discussion

We aimed to determine the acceptability of a proposed practical GA intervention conducted by promotoras for older adult survivors. The authors critically developed a comprehensive understanding of the suitability of community-based geriatric oncology care within an academic, comprehensive cancer center without an existing GA service. Two content categories that we identified, “*The GA intervention employs a motivator to help address survivorship complexities*” and “*The GA intervention plans for impaired ADLs and IADLs in survivorship*” build on the evidence that older adults with cancer are not always receiving any planning or navigation for late and long-term effects following treatments, particularly for physical health declines [27–29]. Other examples of GA-driven supportive care navigation for older adults undergoing treatment have been tested and scaled, such as nurse practitioner-delivered GA (GAIN trial) [30], and remotely delivered cancer survivorship navigation in Brazil [31]. Practical GA is an increasingly critical intervention for clinical oncology with increasing rates of cancer in older adults challenging available specialists [2].

The identified category, “*The GA intervention integrates promotoras with clinical oncology care*” underscores the crucial, collaborative opportunity Promotoras have in geriatric oncology, cancer survivorship, and clinical oncology broadly. Higher rates of cancer incidence and mortality occur in rural and predominantly Hispanic/Latino communities in Northern and Central California (the study catchment area) [32]. Language concordance improves access and adherence to supportive cancer care in male and female cancer survivors [33]. Promotoras work in association with local health care systems [13]. The research team sought their partnership for the future practical GA study (delivered in English and Spanish) given their shared social and cultural backgrounds with the cancer survivor population in the study setting’s catchment area [34].

Together, we identified recommendations for technical infrastructure and the expertise of promotoras to facilitate the practical GA intervention that have been addressed in similar studies. Flexible scheduling and telehealth delivery is suitable and feasible for older cancer survivors seeking supportive care [35]. Promotoras leverage the interconnectedness of communities and systems that are well-suited to cultivate well-being for older adult cancer survivors [36]. Participants seeking supportive care navigation delivered by telehealth report stronger adherence rates and rapport when the navigator shares the local community [37]. The qualitative analysis of these focus groups revealed that promotoras are confident advocates, and appreciate their opportunity to function on behalf of the local, older cancer survivor community within for a busy clinical setting.

There are limitations of this work to consider. The convenience sampling strategy and specific study setting is a concern for reproducibility. However, the study design provided an opportunity to determine acceptability and relevance with community members within the setting of a tentative pilot trial. The research participants in this study agreed to face-to-face interviews to discuss medical topics that other community members may find sensitive. We did not assess feasibility of the trial in this paper, which will be evaluated later using response rates and completion rates [38]. Finally, the open-ended questioning may not have identified challenges with cancer care the participants felt were unrelated to cancer care for older adults.

## Conclusions

The qualitative findings indicate practical GA delivered by promotoras is acceptable for future feasibility testing. Immediate next steps include launching the planned pilot study in collaboration with promotoras to evaluate the feasibility and preliminary effects of practical GA in the investigators’ study institution. We provide a collaborative example of cancer care researchers and community members co-designing supportive care research, with preliminary usability feedback evoked by older cancer survivors.

## Supplementary Information

Below is the link to the electronic supplementary material.

ESM 1 (DOCX 24.5 KB)

## Data Availability

Available on request.
