# Supplementary material for: Acceptability of a practical geriatric assessment intervention with older adult cancer survivors and community health workers/promotoras: a qualitative investigation
Source: Support Care Cancer. 2026 Feb 23;34(3):236. doi: 10.1007/s00520-026-10473-9 (PMC12929298; doi:10.1007/s00520-026-10473-9)
Supplement: Supplementary file 1 — (DOCX 24.5 KB) [file 520_2026_10473_MOESM1_ESM.docx]

|  | Geriatric Oncology Topic: Promotoras | | | |
| --- | --- | --- | --- | --- |
| Concept from Katigbak et al. (2015) Framework | Frailty | Polypharmacy | Symptoms | Caregiver Burden |
| Interpersonal communication techniques to build trust and rapport | How can open and honest communication help the patient understand aging concerns with those around them? (e.g.,.Falls...Needing help w/ cooking/shopping/finances/bathing)  When have you had a good open communication experience about a patient’s age and related issues? | Do you feel comfortable discussing the medications taken and potential concerns about them with the doctor?  How can the doctor create a supportive environment for open discussions about the medications you take? | How can clear and effective communication with your doctors/nurses about cancer symptoms lead to better diagnosis and treatment? | How can open communication with the patients loved one, other family members, and healthcare professionals help in managing caregiver burden?  Share any successful communication strategies that have improved your caregiving experience. |
| Assist with adopting health behaviors (planning; teaching skills; role modeling; enhancing self efficacy) | How can you help older people with cancer pick up healthier habits?  What lifestyle changes have you considered or implemented to improve a patient’s physical and emotional well-being as you (or the patient) ages? | Are you aware of any non-medicated approaches, such as lifestyle changes or complementary therapies like acupuncture, thai chi, yoga, massage, or chiropractor, that could potentially reduce the need for multiple medications?  How open are you to helping the patient explore these alternatives with the doctor? | How can you help older patients with cancer practice healthy behaviors to manage symptoms like pain?  Are you aware of any self-care practices or lifestyle changes that have helped others alleviate cancer symptoms? | Have you talked with any caregiver(s) about having enough support to help the patient during their illnesses? Or in the home? |
| Cultural Congruence with Clients (trusting relationship, shared power gradient) | Have you faced any negative stereotypes or biases when seeking help for age-related challenges?  Do you think having cultural congruence with your provider (ethnicity, language, life experience, etc.) is important for your cancer care?  How can cancer care doctors and nurses better understand your cultural needs when it comes to your care? | Do you have any traditional remedies you use to help manage your health (like teas, herbs, vitamins, etc. )? If so, what are they?  Have your spoken to your doctor about these? | (Left intentionally empty) | In your culture, is caregiving traditionally viewed as a family responsibility? How can cultural norms impact the way caregiving responsibilities are shared or perceived within the family?  How can culture impact the way caregiving responsibilities are shared or looked at within the family? |
| Social Support (information, empathy, reinforcement, access to tools and resources) | How can you help a patient when they are getting progressively weak? E.g., chores/tasks, and go to medical appointments?  How can community and cultural organizations contribute to providing support and resources for older adults with cancer dealing with frailty? (e.g., support groups, community centers, etc.) | How can family members or caregivers help in managing medications, such as organizing prescriptions or assisting with taking your medication? Share any experiences of positive support from loved ones. | (Left intentionally blank) | How can promotoras or caregiver support groups help caregivers cope with the challenges of caregiving?  Share any experiences of receiving valuable assistance or emotional support from your network. |

Opening Qualitative Prompts: Cancer Survivors

1. Thank you for participating. Could you please start by telling us a little about yourself, and what do you remember about planning for your life after completing cancer treatment. What did your oncologist/surgeon tell you to prepare you for this change?
2. What do you remember about your primary care provider telling you about preparing for life after treatment for cancer?
3. What can you tell us about how your oncologist and your primary care provider communicate together? What could have been better in that regard?
4. How often do you see your oncologist/surgeon for follow ups? What do they talk about with you?
5. What has been helpful for you to take care of yourself and any symptoms you may have experienced? What has been helpful for your family caregiver?
6. What resources do you remember were provided to you after you completed treatment? What resources did you find on your own?
7. Have these resources helped you maintain the quality of life you imagined for yourself? If so, how?
8. What can you tell us about nutrition and setting goals for being healthy?

- Follow up about significant weight loss & falls.

1. What resources in the cancer center would you like to see if you have any new challenges? What kinds of roles would you want to interact with? Do you prefer to meet in person or on a video call?

- Follow up about non-licensed health personnel promotoras/CHWs
